# Polyamine Metabolism and Functions: Key Roles in Cellular Health and Disease

**DOI:** 10.3390/biom14121570

**Published:** 2024-12-09

**Authors:** Marianna Nicoletta Rossi, Manuela Cervelli

**Affiliations:** Department of Sciences, University of Roma Tre, 00146 Rome, Italy

The polyamines putrescine, spermidine, and spermine are polycations ubiquitously present in cells, where they exert pleiotropic functions in cellular mechanisms like proliferation, protein synthesis (through the hypusination of the transcription factor EIF5a), redox balance, autophagy, and different forms of cell death [1]. Stabilizing DNA and RNA, polyamines (PAs) play essential roles in cellular growth, gene regulation, and stress responses, making them indispensable in both physiological and pathological contexts [2]. Due to their pleiotropic effects, intracellular PA levels need to be tightly regulated, maintaining a delicate balance between import/export, biosynthesis, and catabolism. Indeed, PA biosynthetic and catabolic enzymes are subjected to stringent control mechanisms operating at various levels, including transcription, translation, and degradation [3,4]. This Special Issue includes nine original research papers and two reviews from experts in the field, providing the reader with knowledge on the advances in the complex and multifaced functions of polyamines in cellular physiology and in different human diseases.

One of the most compelling aspects of polyamines is their role in aging and cellular homeostasis [5]. The decline in polyamine levels with age is associated with increased oxidative stress, reduced autophagy, and impaired cellular repair mechanisms. Spermidine, in particular, has demonstrated potential to mitigate these effects by enhancing cellular resilience and longevity [6,7]. Its ability to promote autophagy and modulate anti-inflammatory pathways underscores the potential for dietary polyamine supplementation to extend health span [8]. On the other hand, a decrease in PA content or an increase in PA catabolism can have deleterious effects [9]. In particular, the enzyme spermine oxidase (SMOX) plays a central role in excitotoxicity by driving oxidative stress through the production of hydrogen peroxide (H_2_O_2_) [10]. This reactive oxygen species (ROS) intensifies neuronal damage during glutamate receptor overactivation, a hallmark of excitotoxicity. Elevated SMOX expression has been associated with heightened neuronal vulnerability to excitotoxic insults [11,12]. In this context, the review by Kashiwagi [13] and Igarashi highlights the role of a metabolite of PA catabolism, acrolein, a reactive aldehyde derived from spermine oxidation [14]. Acrolein is one of the major contributors to oxidative stress [15] and has been found to be more toxic, in aging, than ROS. Acrolein can interact and functionally change several proteins such as MMP9, cytoskeleton proteins, and even glyceraldehyde-3-phosphate dehydrogenase. All these proteins are strongly damaged by acrolein conjugation.

The development of innovative tools to study and visualize intra- and intercellular polyamine trafficking is receiving great attention from the scientific community. In this scenario, the present Special Issue includes a paper from Peter Vangheluwe’s lab describing new fluorescently labeled polyamines and their potential use in the mammalian system [16]. A similar approach has been successfully used to identify a new lysosomal polyamine transporter, ATP13A2, that is mutated in a spectrum of neurodegenerative disorders, including Kufor–Rakeb syndrome and early-onset Parkinson’s disease [17]. Furthermore, research has highlighted a conserved cellular protective mechanism against mitochondrial oxidative stress, facilitated by ATP13A2-mediated lysosomal spermine export [18]. Additionally, work by Khomutov et al. [19] expands the scope of polyamine research by detailing a series of C-methylated spermine analogs. This study explores the biochemical properties of these compounds and their potential to regulate PA activity by altering the position of the methyl group along the polyamine backbone. These derivatives may allow for the study of the interaction of polyamines with OAZ1, a key enzyme in polyamines biosynthesis. Another key biotechnological aspect is the use of protein–nanoparticle hybridization for the treatment of human pathologies, including cancers [20,21]. In this Special Issue, we presented a work where His-tagged spermine oxidase (SMOX) was immobilized for the first time into colloidal surface active maghemite nanoparticles (SAMNs) via direct self-assembly, leading to a biologically active nano-enzyme (SAMN@SMOX). The hybrid was subjected to an in-depth chemical–physical characterization, highlighting that the protein structure was perfectly preserved, and the SMOX catalytic activity toward spermine was even higher at low salinity [22]. The biologically active nano-enzyme SAMN@SMOX holds significant promise as a therapeutic tool, particularly in combination with radiotherapy for cancer treatment. SMOX’s capacity to induce DNA damage has been demonstrated to increase the radiosensitivity of neuroblastoma and fusion-negative rhabdomyosarcoma cells, suggesting its potential to enhance therapeutic efficacy in these malignancies [14,23]. The role of polyamines in cellular proliferation and cancer progression has been long studied [24,25,26]. In the present Special Issue, we included some new findings. In lung cancer cell lines, indomethacin, a common anti-inflammatory drug, increases the spermidine/spermine N1-acetyltransferase 1 (SAT-1) levels, a key component of the PA catabolic pathway. Moreover, the antiproliferative effect of indomethacin can be increased by polyamines oxidase (PAOX) inhibition with methoctramine [27]. Finally, this work highlights the role of the CDK1-nucleolin regulatory axis in this process [28]. Research conducted on breast cancer cell lines and reported in the paper by Van Veen and colleagues established ATP13A4 as a new polyamine transporter and showed that ATP13A4 may play a major role in the increased polyamine uptake of breast cancer cells. Considering that blocking PA uptake has long been considered a way to halt cellular proliferation, ATP13A4 emerges as a candidate therapeutic target for anticancer drugs [29]. Finally, the work by Ivanova and colleagues analyzed the transcriptomic profiles of the hepatoma cell line compared to the liver progenitor cell line, with particular attention paid to changes in the expression of polyamine catabolism genes. The comparison of proliferative hepatoma cells with non-proliferative differentiated non-tumor cells revealed significantly higher levels of the mRNAs of polyamine biosynthesis genes and lower levels of SAT1. This perfectly matches the previous findings of the lower levels of spermine and spermidine in HepaRG cells [30,31].

Targeting the cellular metabolism offers a promising strategy to combat cancer proliferation [32,33]. For example, Sekhar and colleagues tested new methionine depleting compounds in pancreatic cancer cells, showing that methionine depletion reduces intracellular methionine, S-adenosylmethionine (SAM), S-adenosyl homocysteine (SAH), and polyamine levels. Moreover, this treatment induces autophagy. Thus, the ability to modulate the intracellular concentration of methionine represents a new molecular tool used to understand cancer response pathways, including polyamines metabolism [34].

Polyamines are ubiquitous in all living organisms, and this Special Issue provides new perspectives on their metabolism across diverse biological systems, from eukaryotic unicellular organisms to prokaryotes and viruses. In particular, diatoms, efficient carbon capture organisms, contribute to 20% of global carbon fixation and 40% of ocean primary productivity, garnering significant attention to their growth. For the first time, Lin and colleagues described the presence of two spermidine synthase genes and demonstrated that elevated spermidine content promotes diatom growth, providing a foundation for exploring PA functions and regulation in diatoms [35]. In the context of human health, *Streptococcus pneumoniae*, a Gram-positive bacterium, represents a significant threat to human health, causing from mild respiratory infections to severe invasive conditions [36]. The challenges of serotype replacement and antibiotic resistance necessitate alternative therapeutic approaches. In the work from Ayoola and colleagues, two inhibitors of the PA pathways have been tested for their impact on *S. pneumoniae* growth, DFMO that inhibits polyamine biosynthesis, and AMXT-1501 that, targeting polyamine transport, enhances the expression of polyamine biosynthesis. Their findings offer valuable insights into potential therapeutic targets for modulating bacterial growth in a polyamine-dependent manner, a promising avenue for intervention against *S. pneumoniae* infections [37]. Finally, the review from Prof Kaiser recapitulates the impact of spermidine and its metabolites in disease development of the most important, pathogenic human viruses such as SARS-CoV-2, HIV, Ebola, and the human parasites Plasmodium and Trypanosomes. This review also delves into cutting-edge translational approaches for manipulating spermidine metabolism in both hosts and pathogens, offering insights into therapeutic innovation [38].

In conclusion, polyamine research is a rapidly evolving field with significant implications for health and disease. This Special Issue provides comprehensive insights into their multifaceted roles, from mitigating oxidative stress to their applications in cancer therapy. Looking forward, polyamines are poised to become key players in therapeutic strategies, particularly as modulators of cellular metabolism and homeostasis. In conclusion, the landscape of polyamine research is broad and rapidly evolving, with significant implications for health and disease. The present Special Issue offers profound insights into their multifaceted roles, from mitigating oxidative stress to their applications in cancer therapy. Future studies are likely to expand their therapeutic utility, particularly as modulators of cellular metabolism and homeostasis.
